# Supplementary material for: Detecting High-Order Epistasis in Nonlinear Genotype-Phenotype Maps
Source: Genetics. 2017 Jan 18;205(3):1079–88. doi: 10.1534/genetics.116.195214 (PMC5340324; doi:10.1534/genetics.116.195214)
Supplement: Supplementary file 7 [file 1079FileS1.pdf]

Supplement

Zachary R. Sailer[1,2], Michael J. Harms[1,2,*]

1. Institute of Molecular Biology, University of Oregon, Eugene, OR, USA

2. Department of Chemistry and Biochemistry, University of Oregon, Eugene, OR, USA

* harms@uoregon.edu

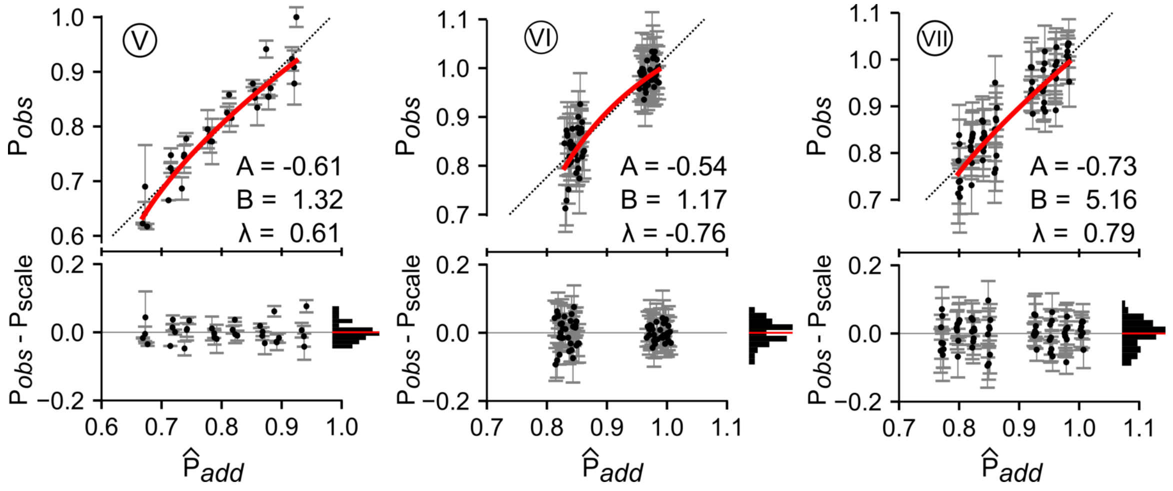

Figure S1: Experimental genotype-phenotype maps exhibit nonlinear phenotypes. Plots show observed phenotype P_{obs} plotted against $\hat{P}_{add}$ (Eq. 1) for data sets V through VII. Points are individual genotypes. Error bars are experimental standard deviations in phenotype. Red lines are the fit of the power transform to the data set. Pearson's coefficient for each fit are shown on each plot. Dashed lines are $P_{add} = P_{obs}$. Bottom panels in each plot show residuals between the observed phenotypes and the red fit line. Points are the individual residuals. Errorbars are the experimental standard deviation of the phenotype. The horizontal histograms show the distribution of residuals across 10 bins. The red lines are the mean of the residuals. Datasets VI and VII form distinct clusters because each map has a single, large-effect mutation. The two clusters correspond to genotypes with and without the large-effect mutation.

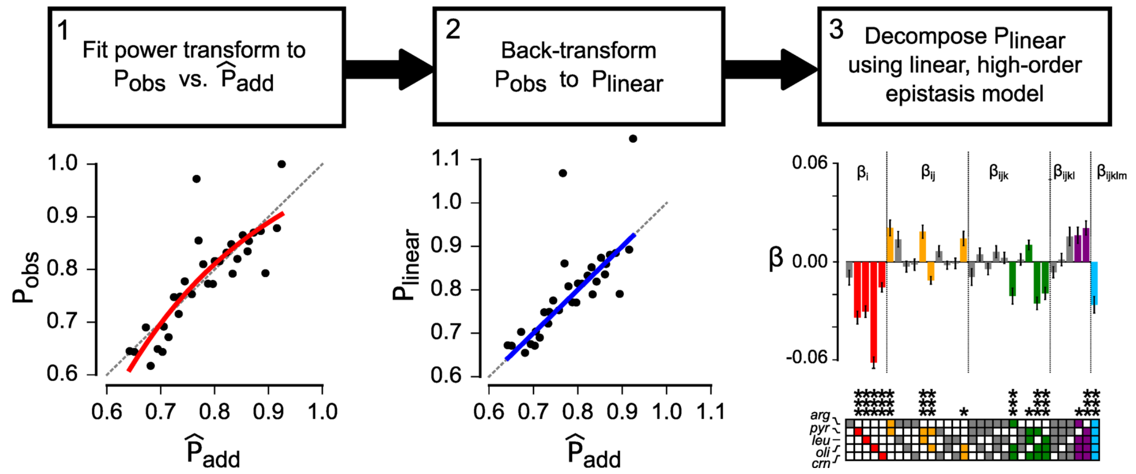

Figure S2: Nonlinear phenotypes can be transformed to linear scale to estimate high-order epistasis. Flowchart shows the steps for estimating high-order epistasis in nonlinear genotype-phenotype maps. The plots beneath the chart show this pipeline for data set II. In step 1, a power transform function is used to fit the P_{obs} versus $\hat{P}_{add}$ plot and estimate the map's scale. In step 2, the inverse of the fitted transform is used to back-transform P_{obs} to a linear scale, P_{linear}. In step 3, a linear, high-order epistasis model is used to fit the variation in P_{linear}. In the left plot, points are individual genotypes, red line is the resulting fit and dashed line is the $P_{add} = P_{obs}$. In the middle plot, the blue line is the new scale of P_{obs} after back transforming. In the right plot, bars represent additive and epistatic coefficients extracted from the linear phenotypes. Error bars are propagated measurement uncertainty. Color denotes the order of the coefficient: first (β_i, red), second (β_{ij}, orange), third (β_{ijk}, green), fourth (β_{ijkl}, purple), and fifth (β_{ijklm}, blue). Bars are colored if the coefficient is significantly different than zero (Z-score with p-value <0.05 after Bonferroni correction for multiple testing). Stars denote relative significance: $p < 0.05$ (*), $p < 0.01$ (**), $p < 0.001$ (***). Filled squares in the grid below the bars indicate the identity of mutations that contribute to the coefficient.

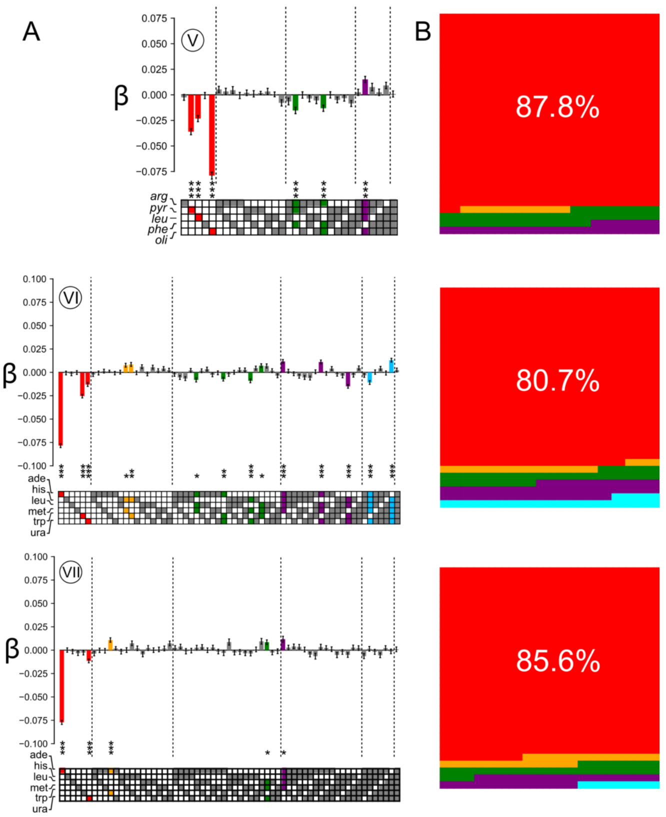

Figure S3: High-order epistasis is present in genotype-phenotype maps. A) Panels show epistatic coefficients extracted from data sets V-VII (Table 1, data set label circled above each graph). Bars denote coefficient magnitude and sign; error bars are propagated measurement uncertainty. Color denotes the order of the coefficient: first (β_i, red), second (β_{ij}, orange), third (β_{ijk}, green), fourth (β_{ijkl}, purple), and fifth (β_{ijklm}, blue). Bars are colored if the coefficient is significantly different than zero (Z-score with p-value <0.05 after Bonferroni correction for multiple testing). Stars denote relative significance: $p < 0.05$ (*), $p < 0.01$ (**), $p < 0.001$ (***). Filled squares in the grid below the bars indicate the identity of mutations that contribute to the coefficient. The names of the mutations, taken from the original publications, are indicated to the left of the grid squares. B) Sub-panels show fraction of variation accounted for by first through fifth order epistatic coefficients for data sets I-IV (colors as in panel A). Fraction described by each order is proportional to area.

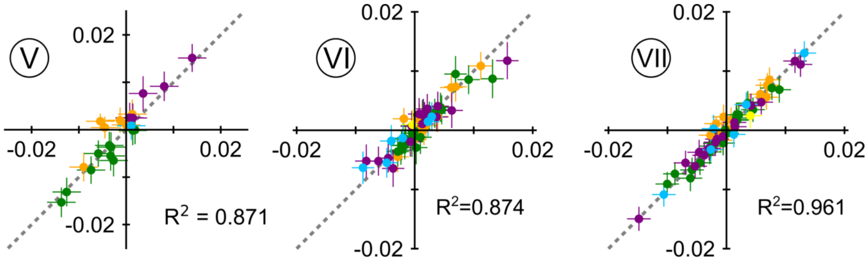

Figure S4: Nonlinear phenotypes distort measured epistatic coefficients. Sub-panels show correlation plots between epistatic coefficients extracted without accounting for nonlinearity (x-axis) and accounting for linearity (y -axis) for data sets V-VII. Each point is an epistatic coefficient, colored by order.

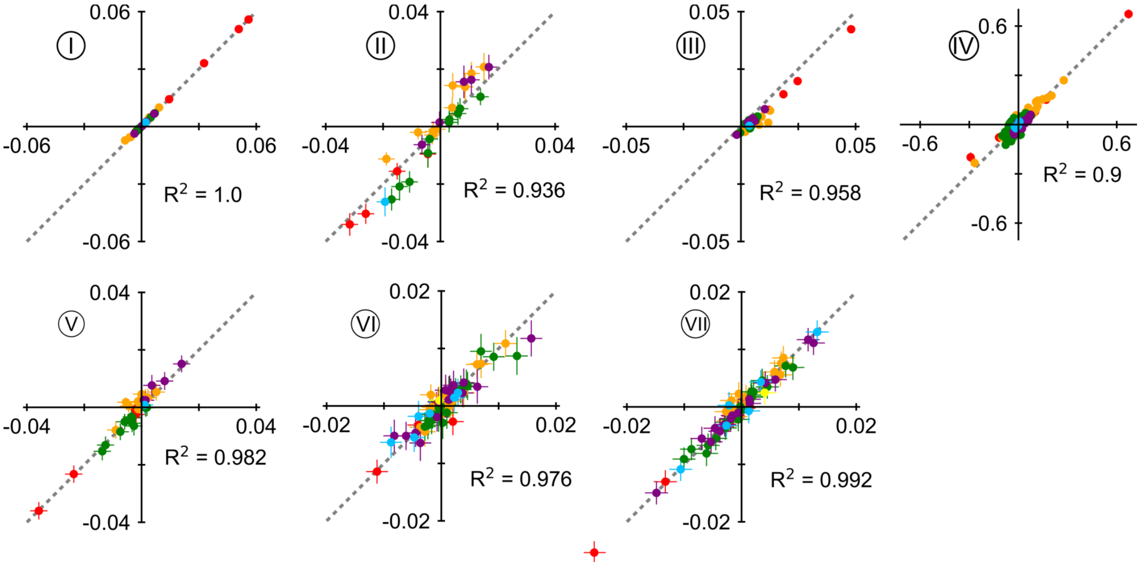

Figure S5: Additive coefficients are well estimated, even when nonlinearity is neglected. Sub-panels show correlation plots between both additive and epistatic coefficients extracted without accounting for nonlinearity (x-axis) and accounting for linearity (y-axis) for data sets I-VII. Each point is an epistatic coefficient, colored by order. Error bars are standard deviations from bootstrap replicates of each fitting approach.

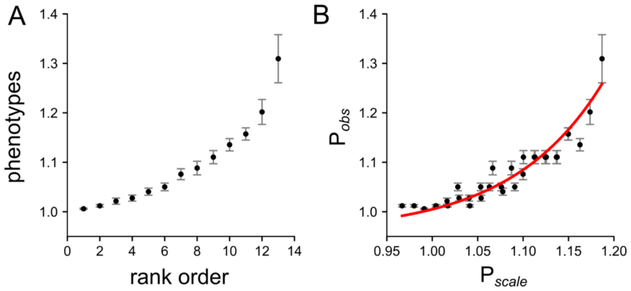

Figure S6: Exponential fitness model leads to global nonlinearity in the β-lactamase data set (III). A) A recapitulation of the map used in the original publication (Weinreich et al. 2006). We first rank-ordered the genotypes according to the measured property (the minimum inhibitory concentration of a β-lactam antibiotic against a clonal population of bacteria expressing that protein). This gave us 13 classes of genotypes, as some genotypes had equivalent MIC values. We then drew 3,000 random fitness values from the distribution $W = 1+x$, where x is an exponential distribution centered around $\bar{x} = 0.1$. We took the top 13 values from this distribution and assigned them, in value order, to each of the 32 β-lactamase genotypes. Panel A shows the average and standard deviation of the fitness values W assigned to each of these ranks if we repeat the protocol above 1,000 times. B) Best fit for the power-transform for data set III. Solid red line denotes the best fit (nonlinear). This fit successfully pulls out the original distribution of W.

References

Daniel M. Weinreich, Nigel F. Delaney, Mark A. DePristo, and Daniel L. Hartl. Darwinian Evolution Can Follow Only Very Few Mutational Paths to Fitter Proteins. *Science*, 312(5770):111–114, July 2006. ISSN 0036-8075, 1095-9203. doi: 10.1126/science.1123539.
